# Super-resolution confocal cryo-CLEM with cryo-FIB milling for *in situ* imaging of *Deinococcus radiodurans*

**DOI:** 10.1016/j.crstbi.2021.12.001

**Published:** 2021-12-13

**Authors:** Danielle L. Sexton, Steffen Burgold, Andreas Schertel, Elitza I. Tocheva

**Affiliations:** a Department of Microbiology and Immunology, University of British Columbia, Vancouver, Canada; b ZEISS Microscopy Customer Center, Oberkochen, Germany

**Keywords:** Cryo-CLEM, Cryo-FIB, Cryo-ET, Cryo-super resolution microscopy, Microbial ultrastructure, Cell envelope architecture

## Abstract

Studying bacterial cell envelope architecture with electron microscopy is challenging due to the poor preservation of microbial ultrastructure with traditional methods. Here, we established and validated a super-resolution cryo-correlative light and electron microscopy (cryo-CLEM) method, and combined it with cryo-focused ion beam (cryo-FIB) milling and scanning electron microscopy (SEM) volume imaging to structurally characterize the bacterium *Deinococcus radiodurans*. Subsequent cryo-electron tomography (cryo-ET) revealed an unusual diderm cell envelope architecture with a thick layer of peptidoglycan (PG) between the inner and outer membranes, an additional periplasmic layer, and a proteinaceous surface S-layer. Cells grew in tetrads, and division septa were formed by invagination of the inner membrane (IM), followed by a thick layer of PG. Cytoskeletal filaments, FtsA and FtsZ, were observed at the leading edges of constricting septa. Numerous macromolecular complexes were found associated with the cytoplasmic side of the IM. Altogether, our study revealed several unique ultrastructural features of *D. radiodurans* cells, opening new lines of investigation into the physiology and evolution of the bacterium.

## Highlights

• User-friendly, commercially available method for correlative cryo-super resolution light microscopy (LM) and cryo-FIB-milling.
• Cryo-super resolution LM, cryo-FIB milling, cryo-SEM volume imaging, and cryo-electron tomography (cryo-ET) to study *Deinococcus radiodurans*.
• Unique *D. radiodurans* cell envelope is composed of two membranes, thick peptidoglycan, an additional layer, and an S-layer.
• Cytoskeletal filaments FtsA and FtsZ were observed at the leading edges of division septa.

## Graphical abstract

**Figure:**
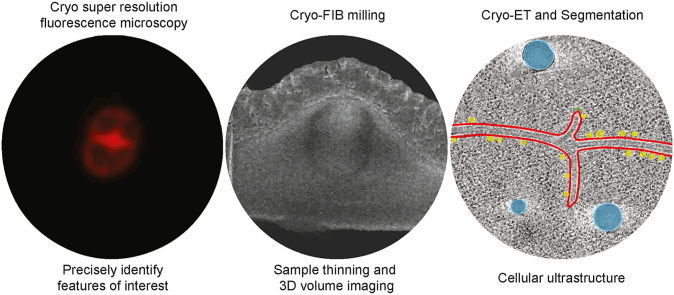

## 1 Introduction

Current advances in sample preparation for cryo-electron microscopy (cryo-EM) allow for the native state preservation and high-resolution imaging of biological specimens. Achieving atomic resolution of individual proteins has helped determine the structure-function relationship of numerous biological targets (Frazier et al., 2020; Herrero Del Valle and Innis, 2020; Davis et al., 2021; Ragonis-Bachar and Landau, 2021). However, due to their inherent thickness, imaging whole cells, especially thick (>0.5 ​μm in diameter) bacteria and eukaryotic cells, has lagged behind. To address this, a combined approach of cryo-focused ion beam (cryo-FIB) milling and cryo-electron tomography (cryo-ET) has been applied successfully to several systems and shows promise for analyzing biological molecules in their native cellular context (Villa et al., 2013; Wagner et al., 2020; Zachs et al., 2020; Klein et al., 2021). In particular, cryo-ET combines two-dimensional (2D) EM with advanced computational analysis to generate three-dimensional (3D) tomographic reconstructions (cryotomograms) of the sample at macromolecular resolution (2–4 ​nm), thereby revealing the complex intracellular architecture of whole cells. However, milling thicker biological samples and identifying proteins of interest within the complex environment of the cell, remain a challenge.

FIB milling and scanning electron microscopy (SEM) tomography is a well-established approach to generate 3D data sets, and was originally used for resin embedded biological samples stained with heavy metals for contrast enhancement (Merchán-Pérez et al., 2009; Narayan and Subramaniam, 2015). This method removes slices of material by FIB milling followed by SEM imaging of the freshly exposed cross-section in a serial manner. This approach was first successfully used under cryogenic conditions (cryo-FIB-SEM) for imaging *Bacillus subtilis* spores and mouse optic nerve tissues (Schertel et al., 2013). Cellular ultrastructure was visualized by detecting low energy secondary electrons without any additional treatment to enhance contrast (Schertel et al., 2013). Subsequently, this method was applied to several biological systems (Steyer et al., 2019; Spehner et al., 2020; Zhu et al., 2021). The large volume cellular context of vitrified biological specimens can be visualized using cryo-FIB-SEM volume imaging, with a slice thickness down to 10 ​nm and a lateral pixel size of >5 ​nm (Vidavsky et al., 2016).

The most powerful method to identify objects of interest in cells for subsequent imaging with cryo-ET is correlative light and electron microscopy (CLEM). In this approach, proteins of interest are fused to fluorophores and coarsely localized using fluorescence light microscopy (fLM). The fLM images are then used to identify targets for subsequent cryo-ET analysis of the same sample. Although powerful, correlating fLM images to cryo-ET has limitations: fLM is best carried out at room temperature using oil-immersion lenses with high numerical aperture (NA ∼1.4). Under these conditions, even slight cellular movement (due to Brownian motion) can prevent subsequent correlation with cryo-ET. As a result, only relatively static structures such as bacterial stalk cross-bands or eukaryotic focal adhesions have been studied (Sartori et al., 2007; Medalia and Geiger, 2010; Schlimpert et al., 2012; Martins et al., 2021). Alternatively, cells can be chemically fixed before imaging. However, fixation can introduce artifacts and destroy intracellular features such as cytoskeletal filaments (Lippincott-Schwartz and Manley, 2009; Schnell et al., 2012; Whelan and Bell, 2015). For best results, prior to fLM imaging cells should be cryogenically preserved either by direct plunge-freezing into liquid nitrogen cooled-ethane (or ethane/propane mixture) or by high-pressure freezing. Preserving the cells cryogenically fixes them in place without introducing artifacts from chemical fixation and allows correlation with subsequent EM approaches (cryo-CLEM). However, cryo-CLEM is severely limited by the fLM resolution because long-working distance air objectives (low NA of ∼0.7) are required for imaging.

To improve the resolution of fLM beyond the diffraction limit of light, significant advances have been made through the development of super-resolution microscopy techniques, including photoactivated localization microscopy (PALM) and structured illumination microscopy (SIM) (Betzig et al., 2006; Gustafsson et al., 2008; Schermelleh et al., 2008). PALM was successfully applied to frozen bacterial samples (cryo-PALM) and cells were subsequently correlated with cryo-ET to reveal the structure of the type VI secretion system in *Myxococcus xanthus* (Chang et al., 2014). Implementation of such approaches, however, requires the use of special photo-activatable molecules, rather than commonly used fluorescent proteins. Additionally, cryo-PALM requires high illumination intensities, which can cause sample devitrification and introduce ice crystals (Phillips et al., 2020). Cryo-SIM was developed to overcome some of the limitations with cryo-PALM, though this technique is not yet integrated with EM techniques (Phillips et al., 2020). Both of these cryo-super-resolution imaging methods rely on the use of custom-built microscopes, which can be challenging to construct and use.

In contrast, the super-resolution Airyscan detector is relatively simple to use and employs a new concept, by imaging an airy disc with a 32-fold GaAsP detector array instead of the conventional single point detector. The Airyscan improves resolution by a factor of up to 1.7 while maintaining the best possible signal-to-noise ratio (SNR), since each of the 32 subunits collects an image simultaneously thus generating a signal 4–8x better than regular detectors (Engelmann et al., 2014). Under cryogenic conditions, the improvement in resolution is closer to 1.5-fold (Rigort et al., 2015). Recently, cryo-Airyscan imaging, cryo-FIB milling and cryo-ET were successfully used to examine the structure of Hps104 aggregates in *Saccharomyces cerevisiae* (Wu et al., 2020).

Here, we describe a workflow that combines the cryo-preservation of biological samples for cryo-ET with the super-resolution capabilities of the Airyscan 2 for cryo-CLEM and cryo-FIB-SEM volume imaging. We use these approaches to structurally characterize the cell envelope of dividing *D. radiodurans* bacterial cells. *D. radiodurans* is a radiation-resistant bacterium with unique structural and functional properties. The bacterium is known to have a unique cell envelope with an outer membrane that lacks the typical lipopolysaccharide (LPS) lipids, and a surface S-layer composed of multiple protein complexes (Farci et al., 2021). The extreme resistance of the *D. radiodurans* cells to UV light and desiccation has been attributed to the cell envelope and in particular the S-layer (Farci et al., 2016). Due to its size, however, direct imaging of *D. radiodurans* with cryo-ET has not been feasible to date. By applying a combination of advanced imaging approaches we were able to reveal the native *in vivo* structure of the cell envelope and division sites of *D. radiodurans*.

Briefly, cells were labeled with FM4-64 membrane dye, plunge frozen on EM finder grids, and identified on a confocal ZEISS LSM 900 microscope equipped with an Airyscan 2 detector. Following transfer onto the ZEISS Crossbeam 550 FIB-SEM, targets were relocated using cryo-CLEM, and 200-nm thick lamellae were generated for subsequent cryo-ET imaging. Cryo-FIB-SEM serial milling and volume imaging were used to target division sites and produced volumes of the surrounding cellular context of the lamella affording additional insights. Lastly, cryotomograms of the lamellae were generated with an FEI Titan Krios operated at 300 ​kV and equipped with a direct electron detector. Our results reveal the ultrastructure of the cell envelope of *D. radiodurans* including the S-layer, and the *in vivo* spatial organization of several subcellular features such as storage granules, macromolecular complexes associated with the inner membrane, and the FtsA/Z cytoskeletal filaments at division sites.

## 2 Materials and methods

### 2.1 Cell culture and plunge freezing

The bacterium used in this study, *Deinococcus radiodurans* BAA-816, was obtained from the ATCC strain collection and grown aerobically in TGY liquid medium (Sweet and Moseley, 1976). Cells were grown for 24 ​h at 30 ​°C prior to harvesting and staining with FM4-64 fluorescent membrane dye (Invitrogen). Four microliters of cells were loaded on Finder EM grids (Electron Microscopy Sciences) and plunge-frozen in a liquid ethane-propane mixture kept at liquid nitrogen temperatures using a Vitrobot Mark IV (Thermo Fisher Scientific). Grids were clipped into AutoGrids™ (Thermo Fisher Scientific) for subsequent cryo-LM imaging and cryo-FIB-SEM milling. Grids were stored under liquid nitrogen and maintained at liquid nitrogen temperatures for the duration of the workflow unless otherwise stated.

### 2.2 Target identification using super-resolution confocal microscopy

Fluorescence maps of vitrified samples on Finder EM grids were acquired using ZEISS LSM 900 confocal microscope equipped with an Airyscan 2 detector, Axiocam 305 camera, a Colibri 7 LED light source for epifluorescence excitation (ZEISS Microscopy GmbH) and CMS196 cryo-stage (Linkam Scientific Inc.). Overview images of the EM grid were acquired in transmitted and fluorescence modes using a 5x NA 0.2 objective lens. Intermediate confocal overview images were collected using a 10x NA 0.4 objective lens in fluorescence and reflection modes. The reflection images were used for grid atlas correlation with SEM images (Fig. S1). To obtain high-resolution fluorescence images of target cells under cryogenic conditions, a ZEISS LD EC Epiplan-Neofluar 100x/0.75 long working distance objective lens was used. Cell morphology could not be discerned when regular confocal imaging mode was used (Fig. S2A). Therefore, Z-stacks were acquired using the Airyscan 2 detector in super-resolution confocal mode with resolution of 290 ​nm in the x- and y-direction, and 1150 ​nm in the z-direction (voxel size of 79 ​nm ​× ​79 ​nm x 470 ​nm and a pixel dwell time of 1.25 μs). Airyscan 3D processing was done to generate the final images and all z-stacks were collapsed to maximum intensity projection (MIP) images (Fig. S2B). The super-resolution z-stack obtained with the Airyscan 2 detector can generate MIP images or 3D models. Since the size of *D. radiodurans* (2–4 ​μm) was close to the z-resolution of the 3D model (1.15 ​μm), we used MIP to locate cells and determine the orientation of division septa. Studies on thicker sample, such as eukaryotic cells, will benefit from the 3D models since the models will provide depth location of a target region. In addition to the improved resolution, the Airyscan 2 detector collected images at high speed and low laser power thus maintaining the sample in a vitrified state. All cryo-fLM data were automatically stored in a ZEN Connect project (ZEISS software for Correlative Microscopy). ZEN Connect aided correlation of different magnifications and imaging modalities by showing an overlay of all the data with the ability to navigate the stage to sites of interest within the correlative workspace.

### 2.3 Cryo-FIB SEM milling of the sample

Cryo-FIB-SEM experiments were conducted on a ZEISS Crossbeam 550 FIB-SEM system (ZEISS Microscopy GmbH) equipped with a Leica VCT500 cryo-stage. In the Leica VCM preparation box (Leica Microsystems), AutoGrids were mounted on a 40° pre-tilted cryo-AutoGrid holder for on-grid thinning (Leica Microsystems). Using the Leica VCT500 Shuttle, the pre-tilted cryo-AutoGrid holder was transferred into the Leica ACE600 cryo-sputter coater (Leica Microsystems) at a cryo-stage temperature of −153 ​°C and samples were sputter-coated with a 6 ​nm thick tungsten layer. The samples were transferred to the ZEISS Crossbeam 550 using the VCT500 Shuttle and a layer of platinum precursor was deposited onto the sample surface in order to minimize the curtaining effect (Zachs et al., 2020). The stage temperature was maintained at −151 ​°C and the system vacuum at 1.4 ​× ​10^−6^ ​mbar pressure. For cryo-FIB-SEM volume imaging and on-grid thinning the stage was tilted to 20°. A stage tilt of 20° resulted in an angle of 16° between the focused ion beam and the 40° pre-tilted grid.

The ZEN Connect project containing the super-resolution confocal Airyscan images of the target cells was imported into the ZEN Connect module of the ZEN Blue software, which was run on a Crossbeam system PC. The imported LM session was aligned with SEM images of the transferred AutoGrid acquired at different stage positions. This resulted in a stage registration which allowed us to use the fluorescence signal to navigate to any target position (Fig. S3). Using image navigation, the stage was positioned at one of the pre-selected targets in the x- and y-direction, and the z-stage position was adjusted such that the center of the SEM image was coincident with the center of the FIB image (the so-called FIB-SEM coincidence point). As a first approach a 12 ​μm wide and 5 ​μm long cross-section ending ∼2 ​μm away from target center was milled from the front side using a FIB current of 700 ​pA (Fig. S4A). Next, using a FIB probe current of 300 ​pA, 20 ​nm thick slices were removed and the freshly exposed cross-section was imaged in a serial manner using InLens SE detection at 2.3 ​kV. For SEM imaging, a probe current of 18 ​pA, dwell time of 100 ns, 5 ​nm lateral pixel size, store resolution of 2048 ​× ​1536, and for noise reduction line average with 37 iterations were used. This live cryo-FIB-SEM serial milling and imaging was used to guide the milling process. When targeted division sites were ∼500 ​nm away from the actual cross-section, the milling with the 300 ​pA probe from the front side was stopped. Milling with the 700 ​pA probe was done from the backside, resulting in an initial lamella of ∼1 ​μm thickness. The lamella was further thinned to about 600 ​nm using the 300 ​pA FIB probe from the back side (Fig. S4B). Next, the lamella was thinned to 400 ​nm from both sides with the 100 ​pA FIB probe. This procedure was followed for all target sites. Finally, all target sites were revisited for final thinning to 200 ​nm from both sides using the 50 ​pA FIB probe. An SEM image of the final lamella was collected for later correlation with cryo-ET (Fig. S4C). The AutoGrid was unmounted and stored in an AutoGrid box under cryogenic conditions.

### 2.4 Cryo-ET, reconstruction and segmentation

The EM grids with milled lamella were transferred to a 300 ​kV Titan Krios transmission electron microscope (TEM) equipped with a Falcon 3 direct electron detector (Thermo Fisher Scientific). Images were acquired using SerialEM software (Mastronarde, 2005). Atlases were collected at 81x to locate the lamellae. Medium magnification images were acquired at 3800x to identify sites for tilt series. Cryo-ET data was collected bidirectionally at 18kx and 22.5kx (pixel sizes 4.6 ​Å and 3.7 ​Å, respectively) with a defocus of 4 ​μm, ±60° oscillation, 1-degree increments with a total final dose of 100 e^−^/Å^2^. Aligned tilt series were CTF corrected using ctfphaseflip before being reconstructed using the weighted back-projection algorithm and SIRT-like filter (SIRT, simultaneous iterative reconstruction technique) in IMOD (Kremer et al., 1996). In addition, Topaz-Denoise algorithm was used to facilitate analysis (Bepler et al., 2020). The density projection profile of the cell envelope was calculated in ImageJ (Schindelin et al., 2012) using a 50 ​nm wide linear section of the cell envelope.

## 3 Results and discussion

**Fig. 1:**
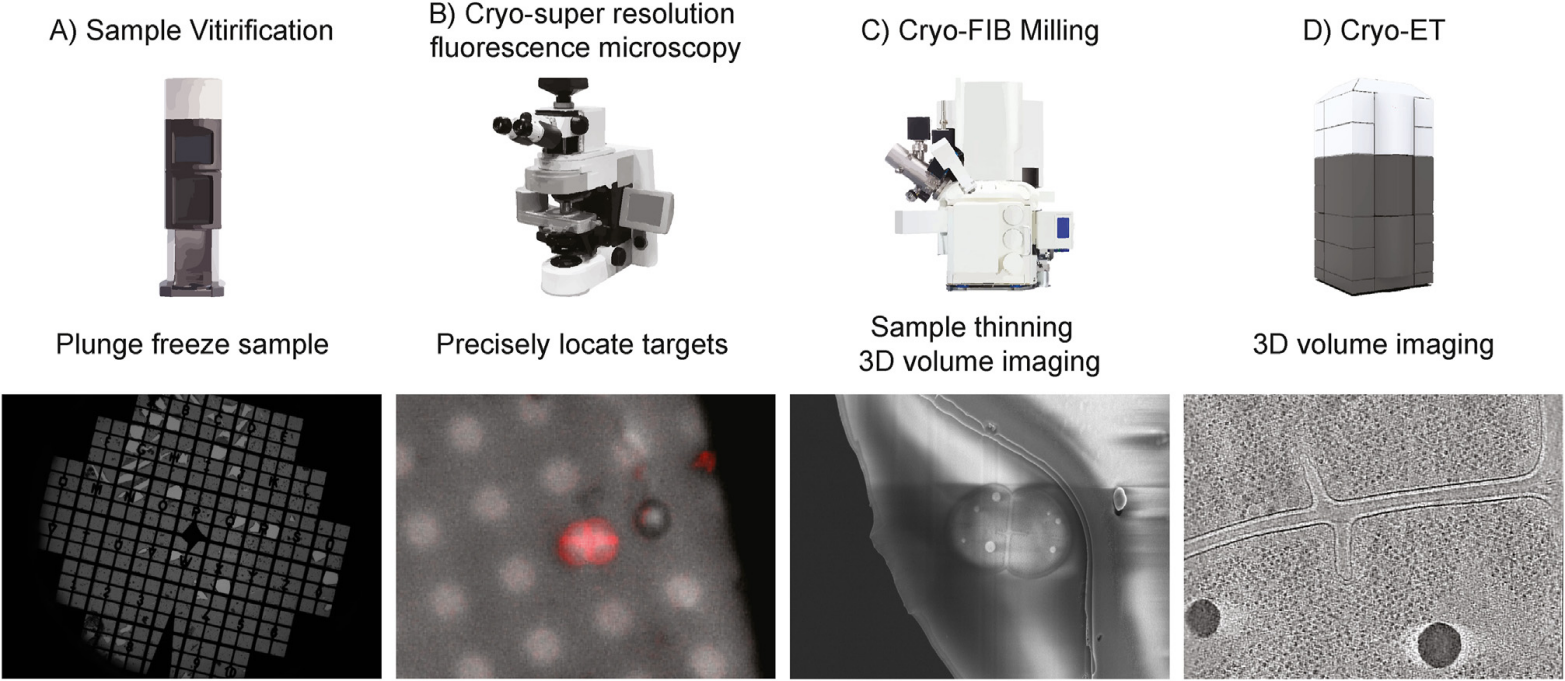
**Cryo-workflow.** A) Bacterial cells were frozen on Finder EM grids with Vitrobot Mark IV. B) Low magnification grid atlases were collected under cryogenic conditions on the ZEISS LSM 900 confocal microscope equipped with an Airyscan 2 detector to identify regions of interest (ROIs). Subsequently, super-resolution fLM images were taken of cells identified in the ROIs. C) The grid was transferred into a cryo-FIB-SEM ZEISS Crossbeam 550 where targets were relocated by correlating SEM with LM images using ZEN Connect. Division sites were targeted using cryo- FIB-SEM volume imaging. 200 ​nm lamellae were milled. D) Cryotomograms of the lamellae were collected on a 300 ​kV Titan Krios TEM.

**Fig. 2:**
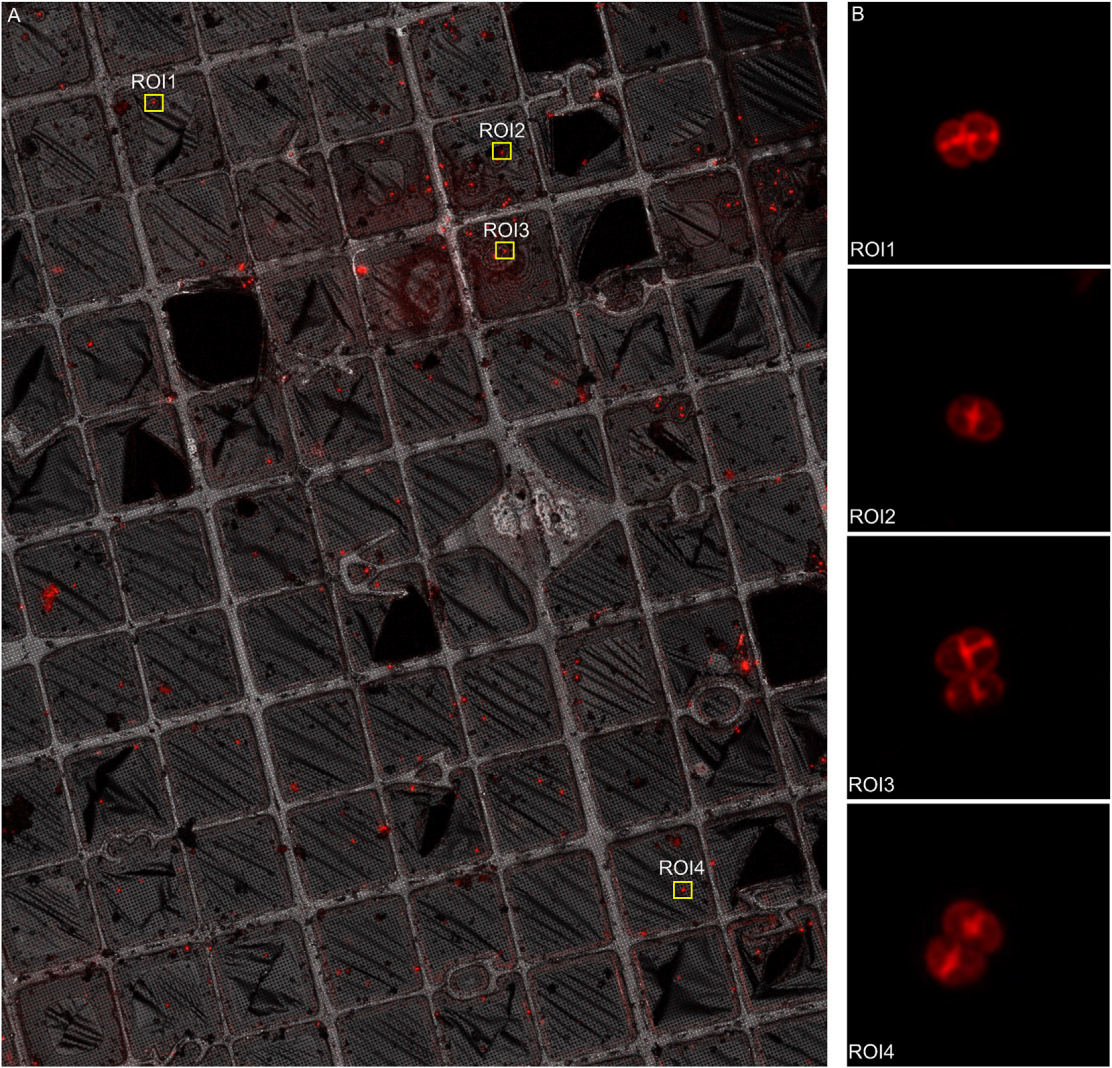
**Fluorescence LM atlas of a Finder EM grid under cryogenic conditions.** Cells were stained with FM4-64 membrane dye, plunge-frozen and maintained cryogenically for imaging. A) A low magnification grid atlas was collected in reflection mode. Regions of interest (ROIs) are boxed in yellow. B) Once targets were identified, z-stacks were collected using the Airyscan 2 detector in super-resolution confocal mode. The maximum intensity projections (MIPs) of four representative z-stacks are shown on the right. Scale bar for A, 100 ​μm. Scale bar for B, 2 ​μm. (For interpretation of the references to color in this figure legend, the reader is referred to the Web version of this article.)

Here we describe the entire workflow of super-resolution confocal cryo-fLM, cryo-FIB-SEM and cryo-ET to accurately map subcellular structures in lamellae of *D. radiodurans* (Fig. 1). Cells were stained with FM4-64 membrane dye and fluorescent images revealed that cells grew in diads and tetrads with an average cell diameter of 2–4 ​μm (Fig. 2B). As such, direct imaging using cryo-ET was not possible and the use of cryo-FIB milling was essential to obtain cryotomograms of *D. radiodurans* cells.

**Fig. 3:**
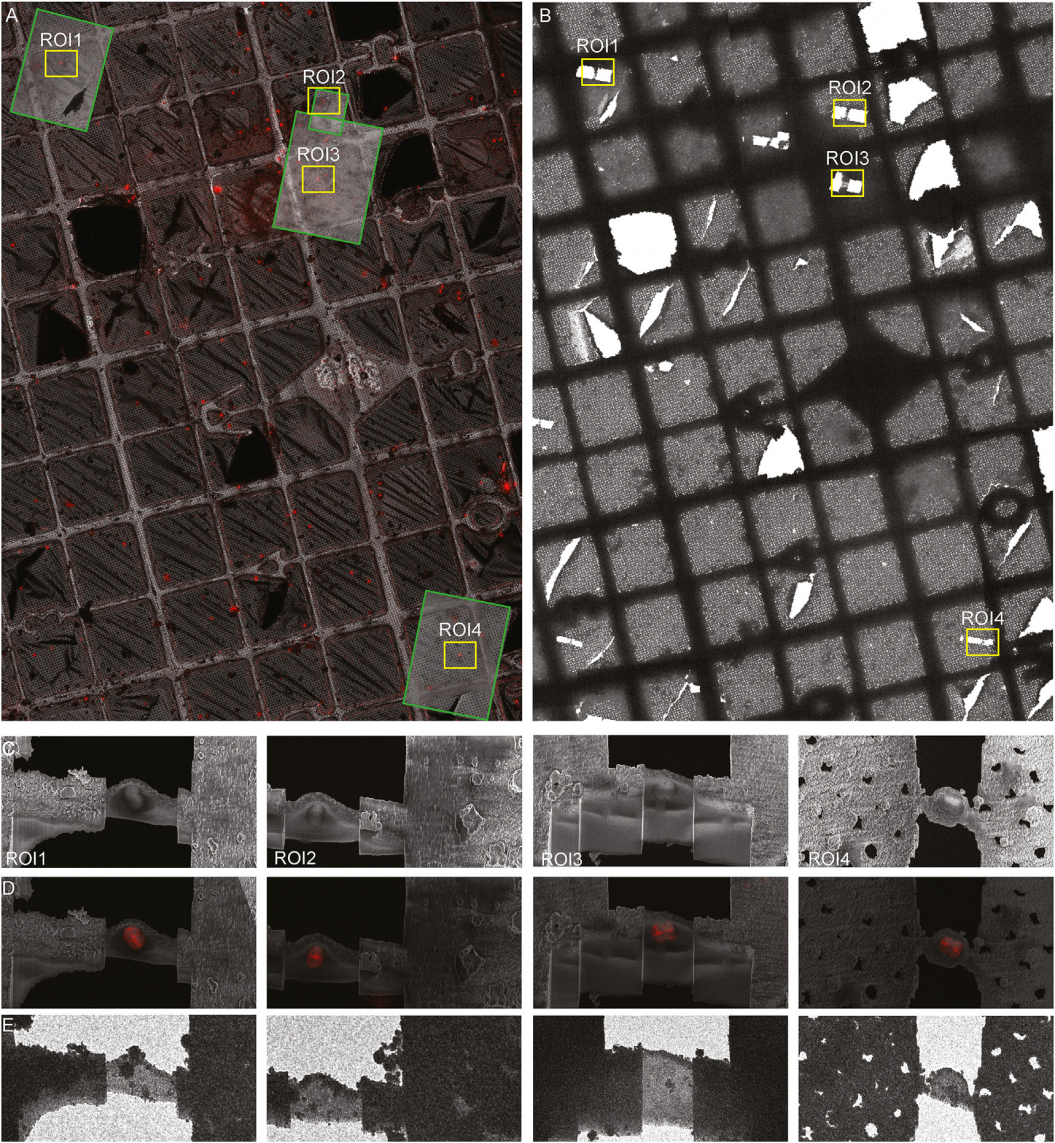
**Correlation and images of final lamellae using fLM, SEM and TEM.** The EM grid was transferred to the ZEISS Crossbeam 550 and targets were relocated by correlating the aligned map of LM images with SEM images. A) After stage registration, medium magnification SEM images (boxed in green) were collected around the targets and were fine-aligned to the corresponding reflection mode images of the LM map in ZEN Connect. Milled areas are boxed in yellow. B) A low magnification TEM grid atlas. Milling patterns and Finder grid features were used for orientation and target identification (yellow boxes). C) Four representative SEM images of final lamellae. D) Correlation of lamellae with the MIP images (FM4-64 channel) from fLM using ZEN Connect. E) Medium magnification images of the lamellae from the TEM used to correlate subsequent cryotomogram collection. Scale bar for A-B, 100 ​μm. Scale bar for C-E, 5 ​μm. (For interpretation of the references to color in this figure legend, the reader is referred to the Web version of this article.)

All LM data in the ZEN Connect project, from super-resolution confocal z-stacks to low magnification LM grid maps, were pre-aligned using their Linkam stage positions. For specific targets, LM images of different magnification were manually fine aligned (Fig. 2A). Positions of regions of interest (ROIs) for subsequent cryo-FIB milling were chosen based on the aligned LM maps (Fig. 2A). The ZEN Connect project from the light microscope was opened in the ZEN Software running on the Crossbeam PC. SEM images were acquired in a new ZEN Connect SEM session and correlated with the ZEN Connect LM session using reflection mode images (Fig. S1). Following correlation, the fluorescent signal was used to navigate to selected target sites in the ZEISS Crossbeam 550 (Figs. 3A and S3). During cryo-FIB milling, volume imaging revealed the precise location and orientation of the target cells (Fig. S4). The super-resolution fLM images and live volume imaging information from the cryo-FIB visually guided the depth of FIB milling to within 500 ​nm of the division planes of the target cells. This correlated approach was used to direct the generation of eight self-supporting lamellae per grid (Fig. 3C and D).

**Fig. 4:**
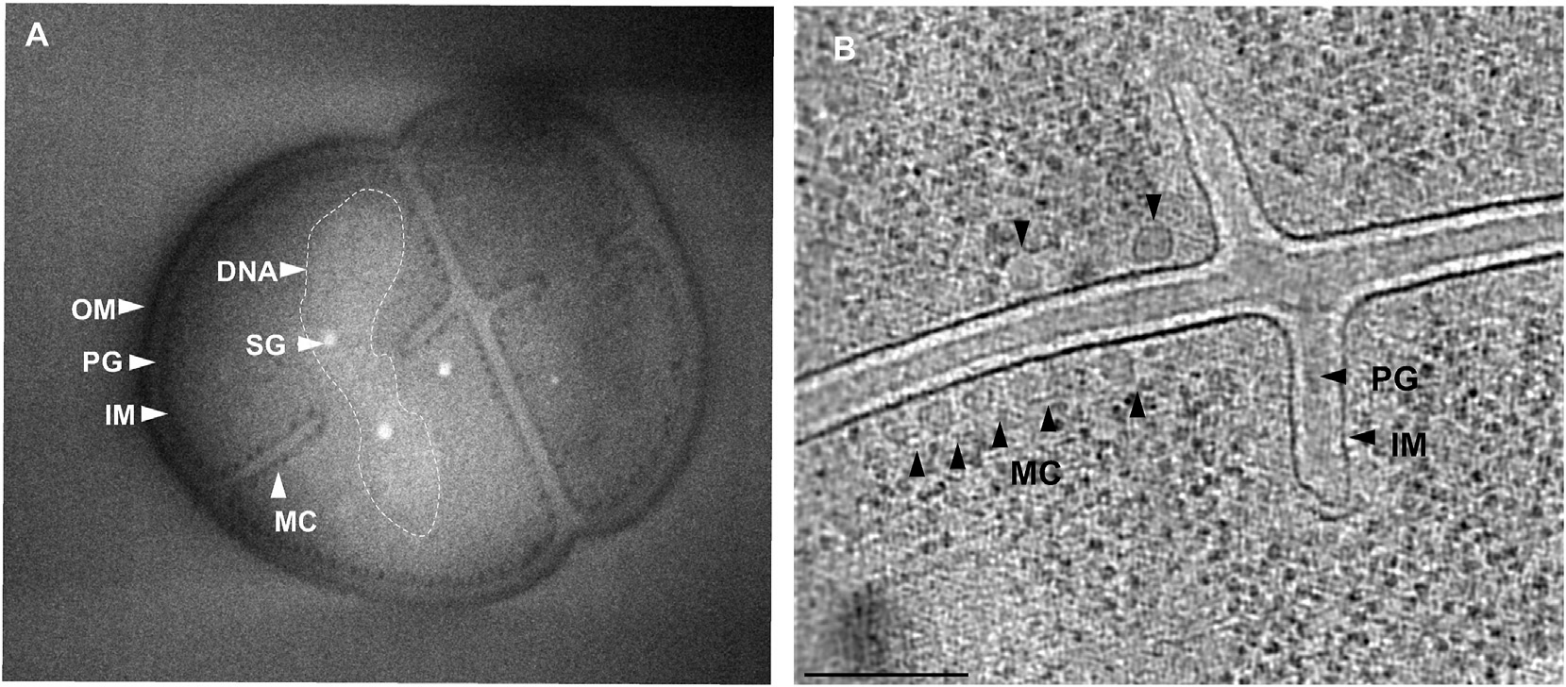
**3D volume imaging vs cryo-ET for detecting subcellular structures.** A) Cryo-FIB-SEM volume imaging revealed the overall cell shape of dividing cells. The cell envelope architecture was poorly discerned. Numerous macromolecular complexes (MC) associated with the IM were clearly visible. Storage granules (SG) were associated with segregating DNA (dashed line). Scale bar, 500 ​nm. B) A slice through the center of a cryotomogram generated using SIRT-like reconstruction and Topaz-Denoise algorithm clearly revealed the PG (∼40 ​nm) and an invaginating IM during cell division. Some MC along the IM (black arrows) were also apparent. Scale bar, 200 ​nm.

Cryo-FIB-SEM volume imaging can be used to: 1) generate volumes of whole cells by serial milling and imaging to reveal subcellular ultrastructure, and 2) provide sequential 2D images of target cells in real-time during FIB milling for targeting the lamella z-position containing the site of interest for cryo-ET (targeting). Knowledge gained from whole cell volume imaging can facilitate feature identification and data interpretation in cryotomograms. For example, the MCs and compacted DNA were more readily observable in cryo-FIB-SEM volumes than in cryotomograms (Fig. 4, Movie S1). The SEM contrast differs from the TEM since the InLens detector captures low energy secondary electrons generated in the focal point providing surface information. In contrast, TEM shows a projection image of the specimen generated by transmitted electrons. For targeting, we used live volume imaging to stop the milling process close to a feature of interest such as the division site of a *D. radiodurans* cell (Fig. S4). In addition, the acquired 3D data stack was used to place tomographic reconstructions into the broader cellular context.

The following is/are the supplementary data related to this article:

movie 12

**Fig. 5:**
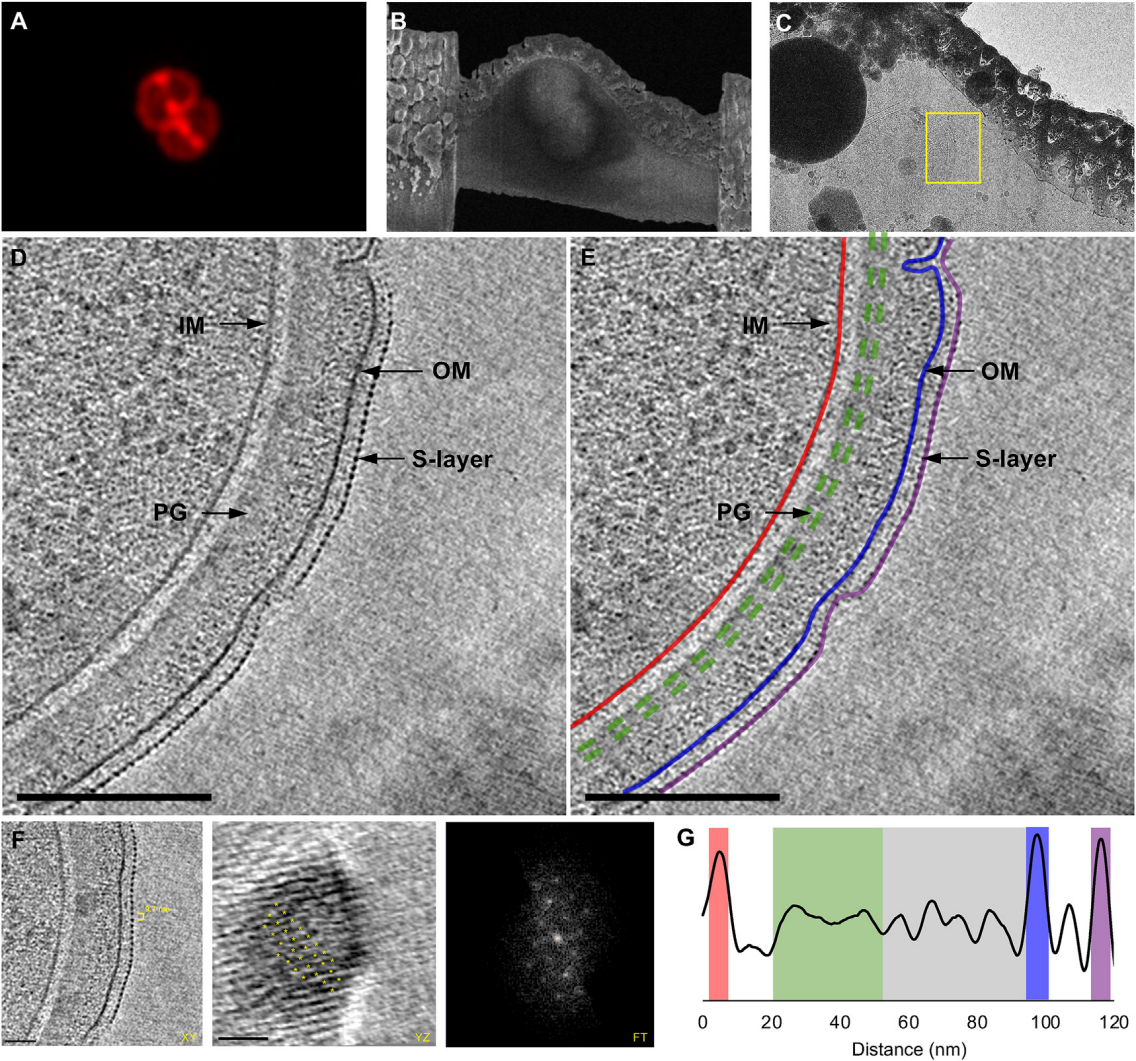
**Cell envelope architecture of *D. radiodurans* revealed by cryo-ET***.* A) Maximum intensity projection image of ROI1 from Fig. 2. Scale bar, 2 ​μm. B) SEM image of the lamella. Scale bar, 2 ​μm. C) TEM image of the lamella with target area boxed in yellow. Scale bar, 1 ​μm. D) 20 ​nm tomographic slice through the target cell showing the inner membrane (IM), outer membrane (OM), surface layer (S-layer), and peptidoglycan (PG). E) Segmentation of the cell envelope showing the IM in red, OM in blue, PG in green, and S-layer in purple. F) Side (XY) and top (YZ) views of the S-layer revealed a hexagonal diffraction pattern (FT) with 9.7 ​nm spacing. G) Density profile of the cell envelope revealed periplasmic space of ∼90 ​nm with ∼40 ​nm thick PG (green), an additional periplasmic layer (gray), and an OM to S-layer distance of ∼20 ​nm. Scale bar, 200 ​nm for D and E; 50 ​nm for F. (For interpretation of the references to color in this figure legend, the reader is referred to the Web version of this article.)

**Fig. 6:**
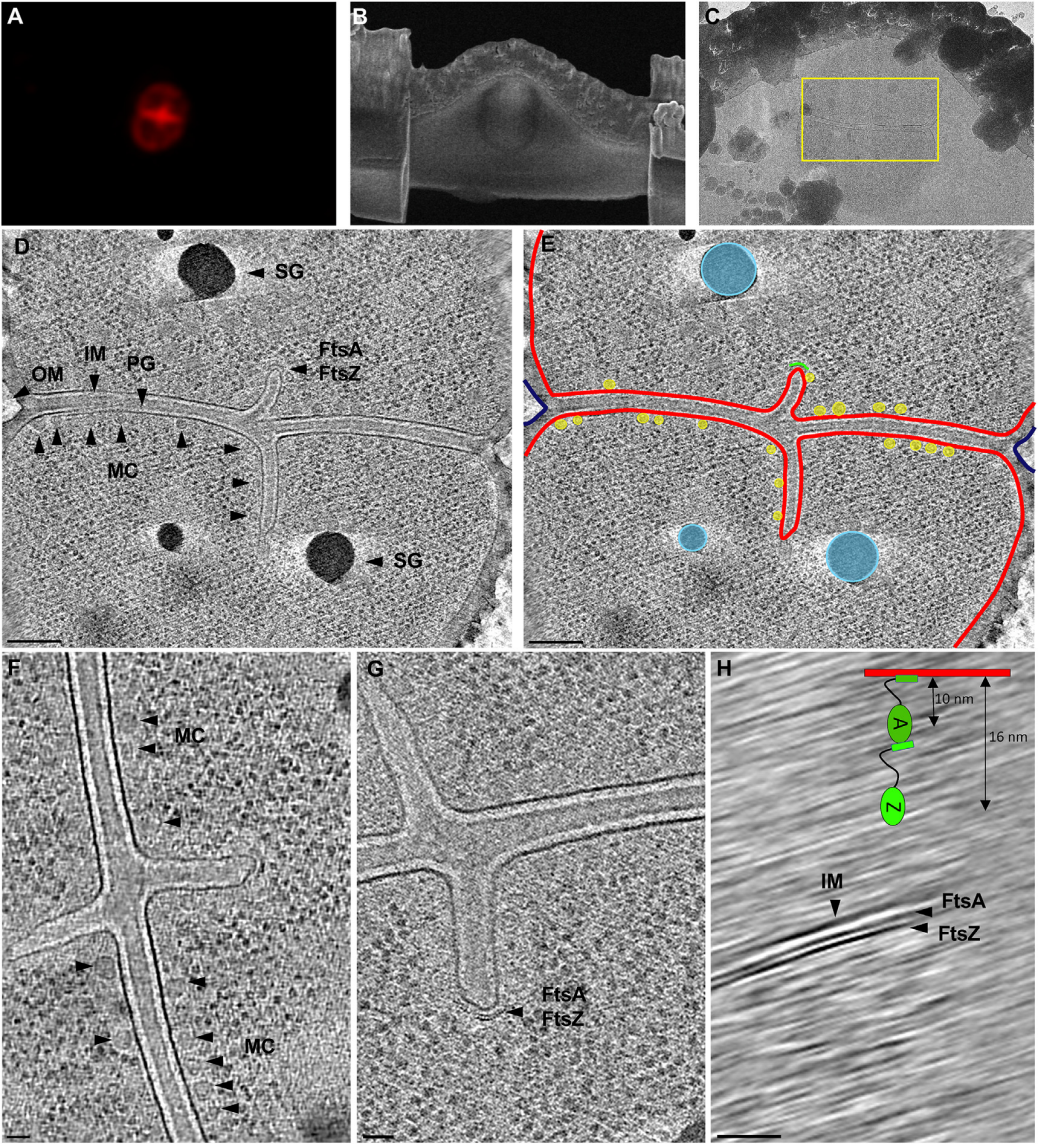
**Ultrastructure of dividing *D. radiodurans* cells revealed by cryo-ET.** A) Maximum intensity projection image of ROI2. Scale bar, 2 ​μm. B) SEM image of lamella post cryo-FIB milling. Scale bar, 2 ​μm. C) TEM image of the lamella with target area boxed in yellow. Scale bar, 1 ​μm. D) 20 ​nm tomographic slice through a target cell showing the inner membrane (IM), peptidoglycan (PG), outer membrane (OM), surface layer (S-layer), storage granules (SG), macromolecular complexes (MC), and FtsA/Z filaments. E) Segmentation of the target cell showing IM in red, OM in blue, SG in light blue, MC in yellow, and FtsA/Z in green. Scale bar, 200 ​nm. F) Images of MC associated with the IM. G) FtsA/Z at the leading edges of constricting IM during cell division. H) YZ view of FtsA/Z filaments along the IM with a model diagram shown in color. Scale bar for F–H, 50 ​nm. (For interpretation of the references to color in this figure legend, the reader is referred to the Web version of this article.)

A grid containing self-supported lamellae (n ​= ​8) was transferred to a 300 ​kV Titan Krios 10.13039/501100001838TEM instrument, where lamellae were identified from the low magnification grid atlas for cryo-ET (Figs. 3B and S1). Reference points from the Finder grid were used to manually correlate the fLM and SEM grid images from the ZEN Connect project with the TEM grid atlas (Fig. 3A and B). Low (Fig. 3E) and medium (Fig. 5, Fig. 6C) magnification TEM images were used to manually correlate the lamella to the other imaging modalities. We collected tilt series on 6 lamellae containing *D. radiodurans* cells, as one lamella was damaged during handling, and another failed to track properly during cryo-ET data collection.

Analysis of the cryotomograms revealed a unique multilayered cell envelope architecture with a clear IM, thick layer of PG, an OM, and a prominent surface layer (S-layer) (Fig. 5D, E, G). Typical distance between the IM and OM of diderm bacteria is ∼30 ​nm, however, the observed periplasmic space in *D. radiodurans* was unusually large: ∼90 ​nm (Fig. 5G). In addition, the PG layer of typical diderm cells is thin (<10 ​nm), whereas the PG in *D. radiodurans* was significantly thicker (∼40 ​nm). The PG appeared continuous, suggestive of uniform PG composition. An additional layer with granular appearance was observed between the PG and OM. The composition of this layer is unknown, however, the PG and the additional granular layer appeared very similar to the inner and outer cortex layers found in bacterial endospores (Tocheva et al., 2013b). The cortex in endospores is modified PG and provides extreme resistance to heat, desiccation, UV and γ-radiation (Beskrovnaya et al., 2021). Interestingly, during cell division, only the PG layer (40 ​nm thick) was observed between the leading edges of constricting IMs, further suggesting that the role and structure of the two periplasmic layers is different (Fig. 6). The presence of a thick PG layer and possibly a modified cortex layer in *D. radiodurans* is unusual for a diderm bacterium and might be particularly important for resistance to radiation and desiccation.

Previous studies have shown that the S-layer in *D. radiodurans* is critical for resistance to UV stress and contributes to heat tolerance (Farci et al., 2016, 2018, 2020), making it an important structure for the overall resistance of *D. radiodurans* to environmental stresses. This layer was observed 20 ​nm above the surface of the OM (Fig. 5). The S-layer in bacteria is often composed of one protein with a conserved S-layer homology domain (SLH). Recent studies on the S-layer in *D. radiodurans* show that it is composed of three different protein complexes: the S-layer deinoxanthin-binding complex (with SlpA as the main component), a type IV-like piliation system, and a dihedral complex (Farci et al., 2021). Based on cryo-EM of purified S-layers, the proteins were shown to form paracrystalline sheets with hexagonal symmetry and 9.6 ​nm lattice spacing (Baumeister et al., 1982; Farci et al., 2015, 2021). The power spectrum of the S-layer from our cryotomograms confirmed the hexagonal pattern with 9.7 ​nm spacing (Fig. 5F), and further revealed its association with the cell envelope.

The OM of *D. radiodurans* lacks LPS and phospholipids that are typically found in the outer leaflet of OMs in Gram-negative bacteria, and instead has a similar lipid composition as the IM (Thompson and Murray, 1981). Our cryotomograms showed that the IM and OM were ∼7 ​nm thick, consistent with a lipid bilayer (Fig. 5D, E, G), though, the OM appeared slightly denser than the IM (Fig. 5G). During cell division, the septum formed by the invagination of the IM, followed by a thick layer of PG (Fig. 6F and G). Clear filaments were observed at the leading edges 10 and 16 ​nm from the IM (Fig. 6G and H). The spacing and positioning of the filaments is consistent with FtsA and FtsZ shown to drive septa constrictions in other bacteria (Jensen et al., 2005; Li et al., 2007; Szwedziak et al., 2014). Upon septation, the OM invaginates and completes cell division. Storage granules, likely composed of polyphosphate (Tocheva et al., 2013a), were observed in the cells, with diameters ranging from 20 to 150 ​nm (Fig. 6D and E). These could serve as a phosphate source for the production of phosphate-manganese complexes used by the cell to protect macromolecules from oxidative damage, in addition to serving as building blocks for cellular components (Daly et al., 2010; Slade and Radman, 2011; Bruch et al., 2015; Santos et al., 2019). Numerous macromolecular complexes (MCs) ∼30 ​nm in diameter appeared near or associated with the IM on the cytoplasmic side (Fig. 4, Fig. 6F). The size and distribution of these complexes was similar to previously-described clusters of Dps2, which bind and oxidize iron to protect cellular contents from oxidation (Santos et al., 2019). The MCs were not readily observable in cryotomograms suggesting that they might not be associating with high levels of iron, however, they were clearly visible in cryo-FIB-SEM images (Fig. 4). Future studies identifying these abundant complexes will reveal more information about their composition and physiological role.

Overall, our study revealed the *in vivo* architecture of the cell envelope in *D. radiodurans*, as well as the ultrastructure of division sites. Cytoskeletal filaments, storage granules and MCs are observed to nanometer resolution in the context of the cell. In addition, future studies characterizing the unique PG, novel periplasmic layer, and complex S-layer will provide insights into their molecular arrangements and role in extreme resistance to oxidation and irradiation.

## CRediT authorship contribution statement

**Danielle L. Sexton:** prepared samples for cryo-CLEM, collected tilt series, reconstructed tomograms., performed denoising and segmentation and prepared figures, wrote the manuscript with support from SB. **Steffen Burgold:** performed cryo-fLM imaging. **Andreas Schertel:** performed cryo-FIB-SEM imaging and TEM lamellae preparation, data processing and figures, wrote the manuscript with support from SB. **Elitza I. Tocheva:** conceived and designed the experiments, prepared samples for cryo-CLEM, collected tilt series, reconstructed tomograms, wrote the manuscript with support from DLS and SB.

## Declaration of competing interest

The authors declare that they have no known competing financial interests or personal relationships that could have appeared to influence the work reported in this paper.
