# Supplementary material for: Super-resolution confocal cryo-CLEM with cryo-FIB milling for in situ imaging of Deinococcus radiodurans
Source: Curr Res Struct Biol. 2021 Dec 13;4:1–9. doi: 10.1016/j.crstbi.2021.12.001 (PMC8688812; doi:10.1016/j.crstbi.2021.12.001)
Supplement: Multimedia component 1 [file mmc1.pdf]

SUPPLEMENTAL MATERIAL

Super-resolution confocal cryo-CLEM with cryo-FIB milling for *in situ* imaging of *Deinococcus radiodurans*

Danielle L. Sexton[1], Steffen Burgold[2], Andreas Schertel[2], Elitza I. Tocheva[1,*]

[1]Department of Microbiology and Immunology, University of British Columbia, Vancouver, Canada

[2]ZEISS Microscopy Customer Center, Oberkochen, Germany

[*]Correspondence to: elitza.tocheva@ubc.ca

Supplemental Movie S1: Cryo FIB-SEM volume imaging of *D. radiodurance* cells. Cryo-FIB-SEM volume imaging parameter: FIB Milling: 300 pA at 30 kV. 20 nm slice thickness, 75 slices in total. SEM Imaging: 2.3 kV EHT, I Probe 27 pA, InLens SE detector, 5 nm lateral pixel size.

Supplemental Figures:

Figure S1: Coarse grid atlas registration for confocal LM, cryo-FIB, and cryo-ET imaging.

Figure S2: Comparison of regular vs super-resolution confocal imaging.

Figure S3: Corelative light and electron microscopy used to guide cryo-FIB milling.

Figure S4: Sequential cryo-FIB-SEM milling patterns for lamella generation.

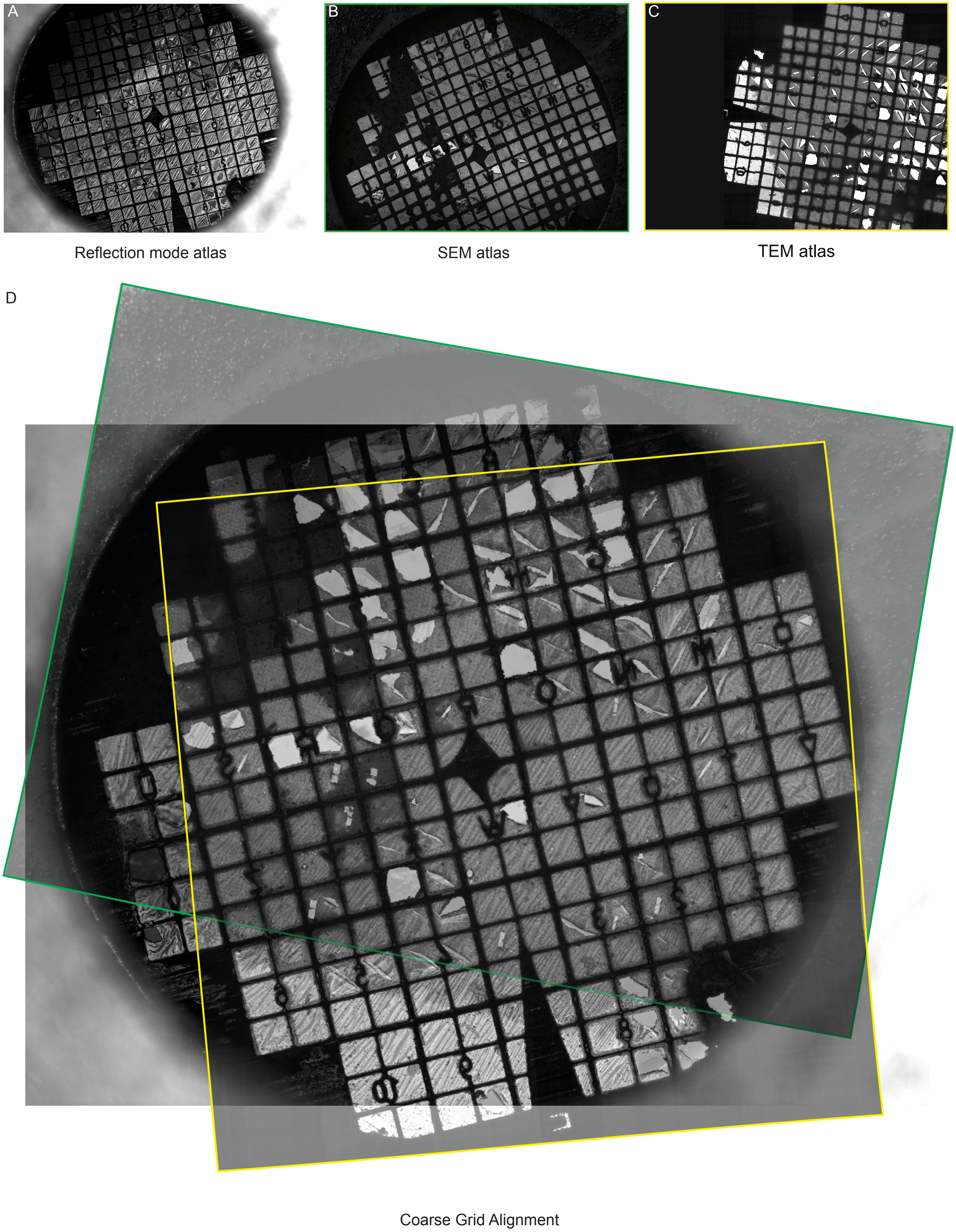

Fig. S1 Coarse grid atlas registration for confocal LM, cryo-FIB, and cryo-ET imaging. A) An overview of the grid was collected at 5x magnification in reflection mode on the confocal microscope. B) After the grid was transferred to the FIB-SEM, a second grid atlas was collected using a series of low magnification images in SEM mode. C) After the grid was transferred to the TEM, a series of low magnification images was collected to produce a grid map. D) The SEM atlas (outlined in green) was coarsely aligned to the reflection mode atlas to begin stage registration in ZEN Connect. The TEM grid atlas (outlined in yellow) was coarsely aligned with the reflection mode and SEM atlas to identify lamellae and direct tilt series data collection.

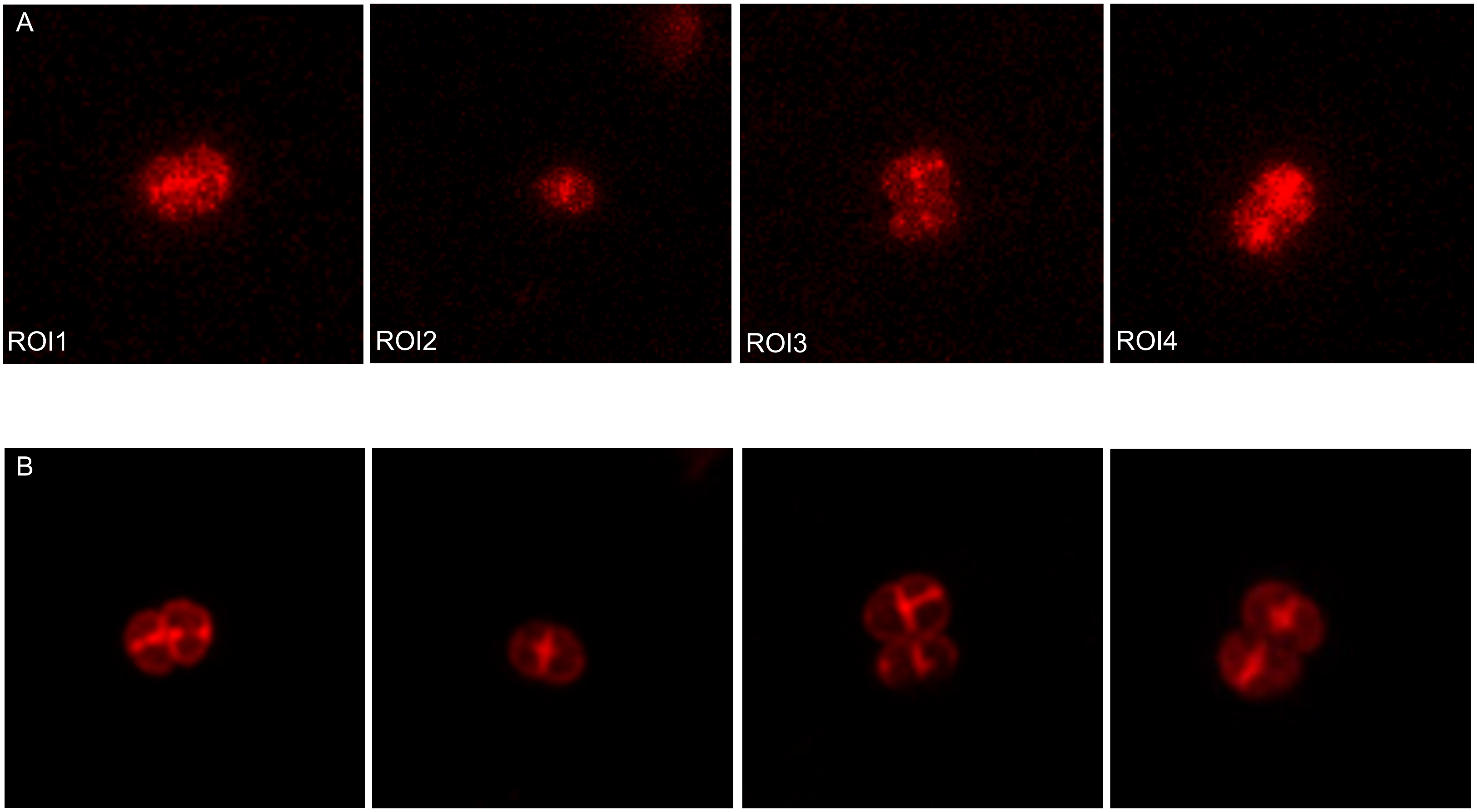

Fig. S2. Comparison of regular vs super-resolution confocal imaging. Cells were stained with FM4-64 membrane dye and imaged under cryogenic conditions. A) MIPs generated with regular confocal imaging, and B) MIPs generated with super-resolution confocal imaging using the Airyscan detector. Images from B were used to identify targets for subsequent cryo-FIB milling.

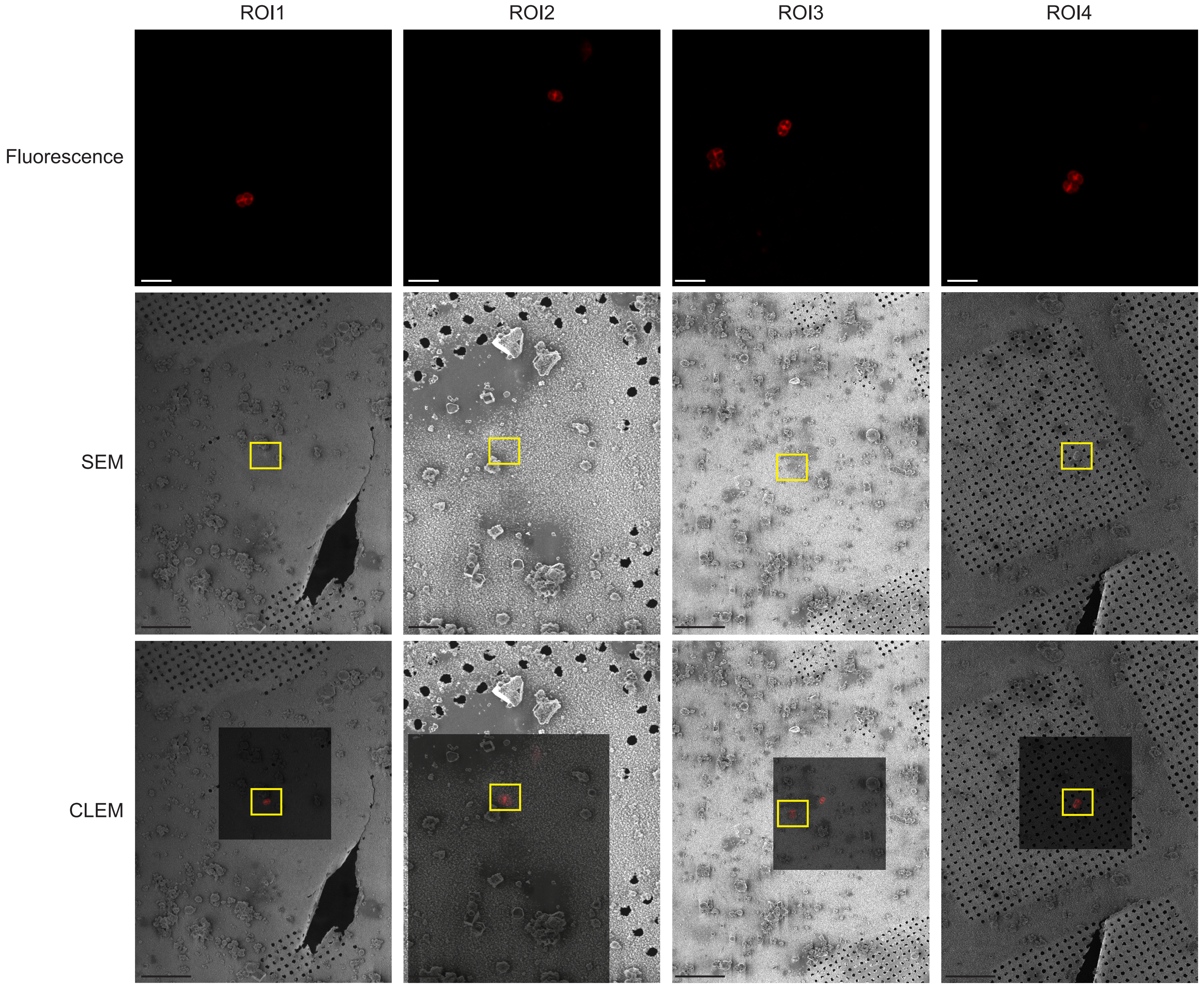

Figure S3: Corelative light and electron microscopy used to guide cryo-FIB milling. Medium magnification SEM images were collected at each position prior to FIB milling and were correlated with the aligned LM map using reflection mode to relocate the targets in the ZEISS Crossbeam 550. Superposition of the fluorescent images with the SEM images indicated precise target location. Milled targets are highlighted with yellow boxes for each ROI.

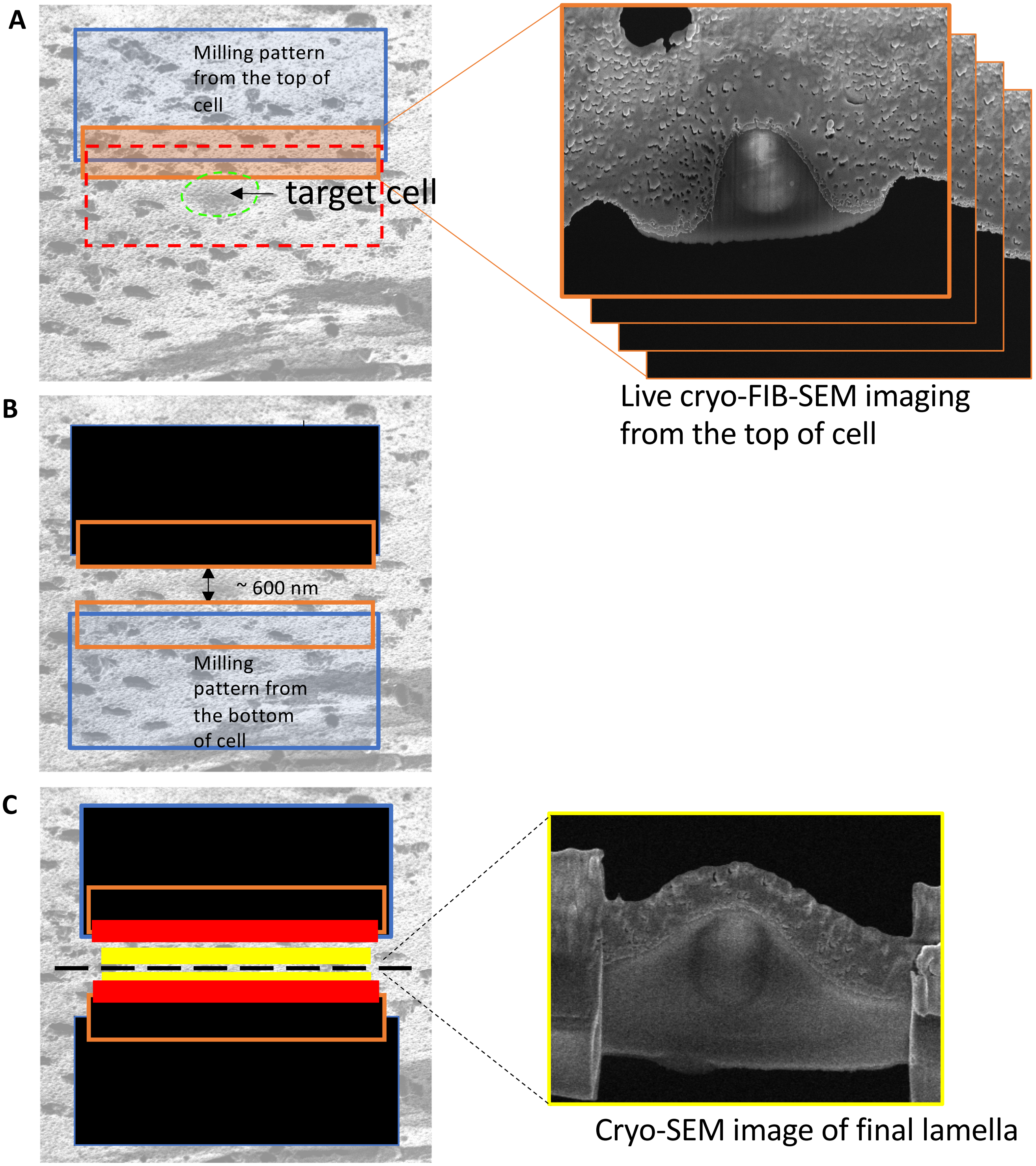

Fig. S4. Sequential cryo-FIB-SEM milling patterns for lamella generation. A) The grid was loaded in the Crossbeam 550 with the bacterial cells facing upwards. Initial coarse cross-sectioning using 700 pA (blue rectangle) approached the target cell (green dotted circle) by ~2 µm from the top side. Next, the area shown in red dotted rectangle was used to determine the serial sectioning and imaging pattern for the 300 pA probe. Live cryo-FIB-SEM imaging (shown on the right) was used to guide the process. When division sites were approached by ~500 nm, the milling process was stopped. Orange rectangle shows the final area milled with the 300 pA probe. B) Using the 700 pA and 300 pA probes, the cell was milled from the bottom resulting in a 600 nm thick lamella. Black rectangles represent already milled areas shown in A. C) Using the 100 pA probe, the volume was thinned from both sides to final thickness of ~400nm (red rectangles). This procedure was followed for all target sites. Lastly, a 50 pA probe was used to thin the sample to the final lamella thickness ~200 nm (yellow rectangles). Dashed line represents the position of the final lamella (shown on the right).
